# Case report: Non-surgical triumph in pericardial paraganglioma: durable tumor regression via targeted embolization of coronary collaterals

**DOI:** 10.3389/fonc.2026.1751548

**Published:** 2026-05-11

**Authors:** Zaiqiang Zhang, Jiawang Ding

**Affiliations:** 1 Department of Cardiology, The First College of Clinical Medical Sciences, China Three Gorges University, Yichang, Hubei, China; 2 Institute of Cardiovascular Diseases, China Three Gorges University, Yichang, Hubei, China

**Keywords:** case report, imaging, multimodal, paraganglioma, pericardial, transcatheter embolizatio, tumor

## Abstract

**Background:**

Pericardial tumors are rare cardiovascular disorders that often present with non-specific clinical symptoms and have traditionally required surgical intervention. Multimodal imaging is crucial for diagnosis, treatment planning, and prognostic evaluation.

**Case presentation:**

A 60-year-old woman was referred to our institution with a one-year history of progressive exertional chest tightness and dyspnea, which had significantly worsened over the preceding month. Multimodal imaging diagnosed a pericardial tumor dependent on a circumflex artery fistula for its blood supply. Transcatheter embolization of the fistula was consequently performed to devascularize the tumor. At the 1-year follow-up, surveillance imaging demonstrated significant tumor regression. During follow-up, the patient reported substantial resolution of symptoms, and her blood pressure remained well-controlled.

**Conclusion:**

This case highlights the critical role of multimodal imaging in diagnosing and managing pericardial tumors. Furthermore, it demonstrates the therapeutic potential of transcatheter embolization for inoperable patients with pericardial paragangliomas.

## Background

Pericardial tumors represent an exceedingly rare group of cardiovascular disorders. Epidemiological studies indicate that the incidence of primary pericardial tumors ranges from 0.001% to 0.007% (1). According to Meng and Patel primary pericardial tumors account for only 6.7% to 12.8% of all primary cardiac tumors (2). Given their nonspecific clinical manifestations during early disease stages, imaging examinations play a crucial role in diagnosis, treatment planning, and prognostic evaluation. We present a distinctive case of a pericardial tumor supplied by coronary collateral vessels. Following interventional embolization of the feeding vessels, the tumor demonstrated significant regression, and the patient’s abnormal hormone levels normalized during the 1-year follow-up period.

## Case presentation

**Figure 1:**
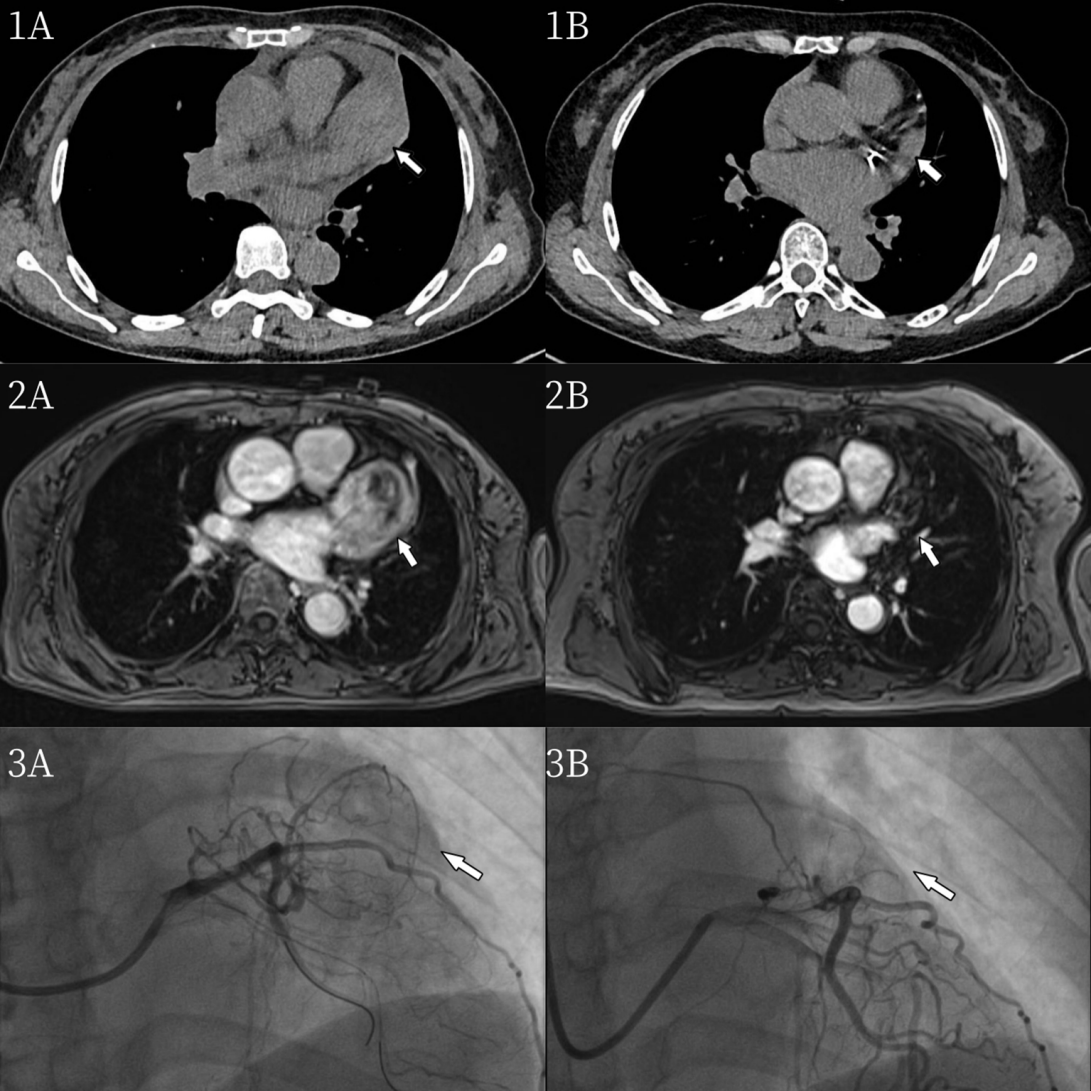
**(A)** Chest CT revealed a soft tissue tumor of approximately 50×40×30 mm in the pericardial region superior to the left ventricle. **(B)** One year after interventional embolization, the pericardial tumor significantly decreased in the Chest CT. **(A)** Cardiac MRI confirmed a pericardial tumor in the basal left ventricular region. **(B)** One year after interventional embolization, the pericardial tumor significantly decreased in the Cardiac MRI. **(A)** Coronary angiography confirmed a fistulous tract from the circumflex artery supplying the pericardial tumor. **(B)** Coronary angiography confirmed the pericardial tumor significantly decreased.

**Figure 2:**
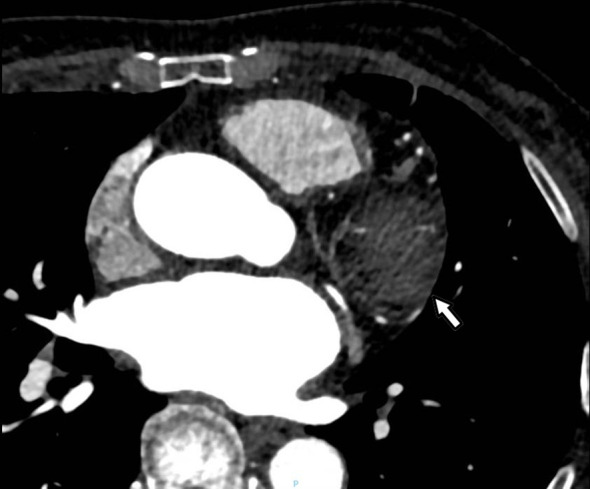
Coronary CTA confirmed a neoplastic pericardial tumor with a suspected coronary blood supply.

**Figure 3:**
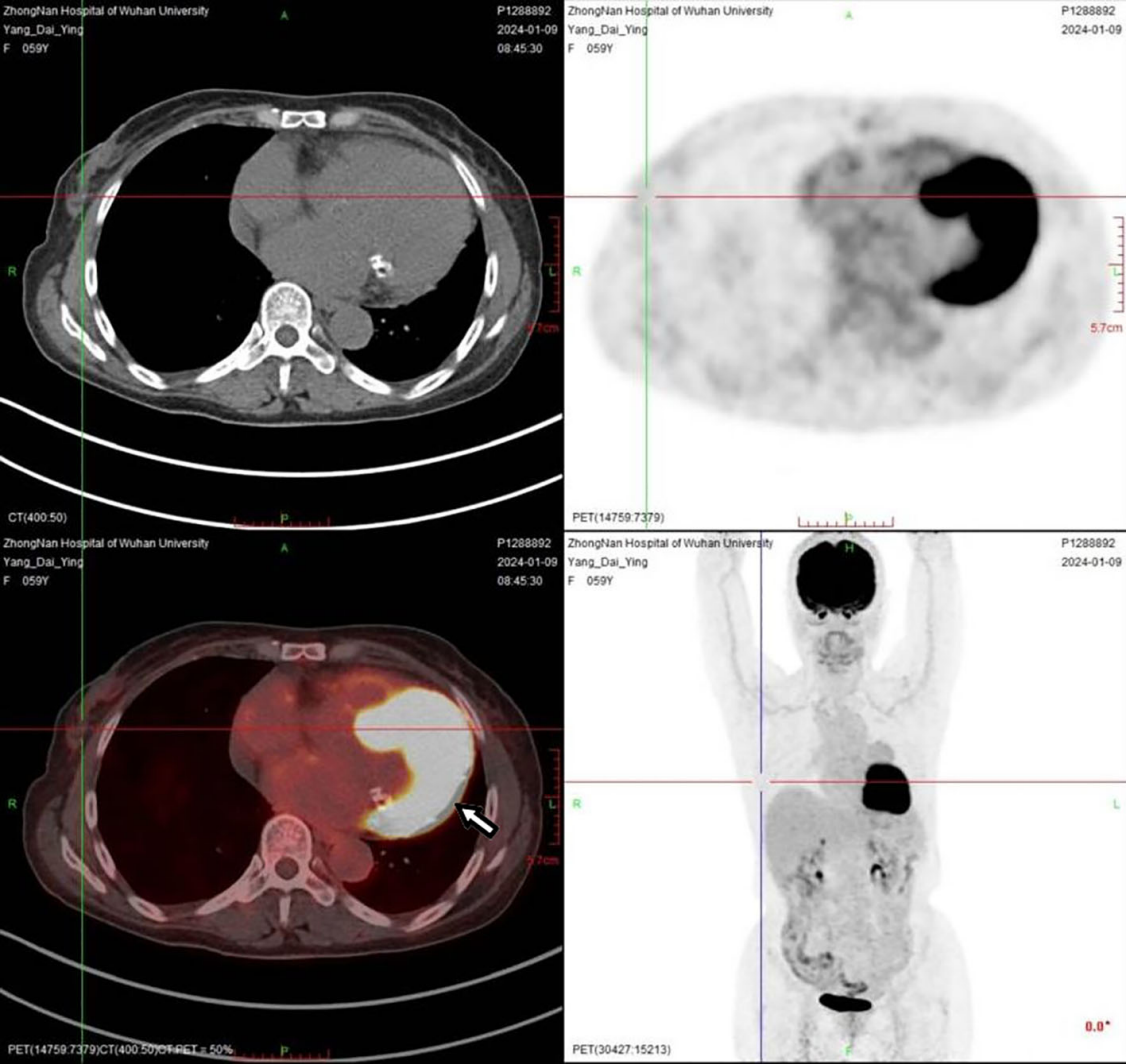
FDG-PET demonstrated heterogeneous fluorodeoxyglucose uptake within the tumor, suggestive of a malignant neoplasm.

**Figure 4:**
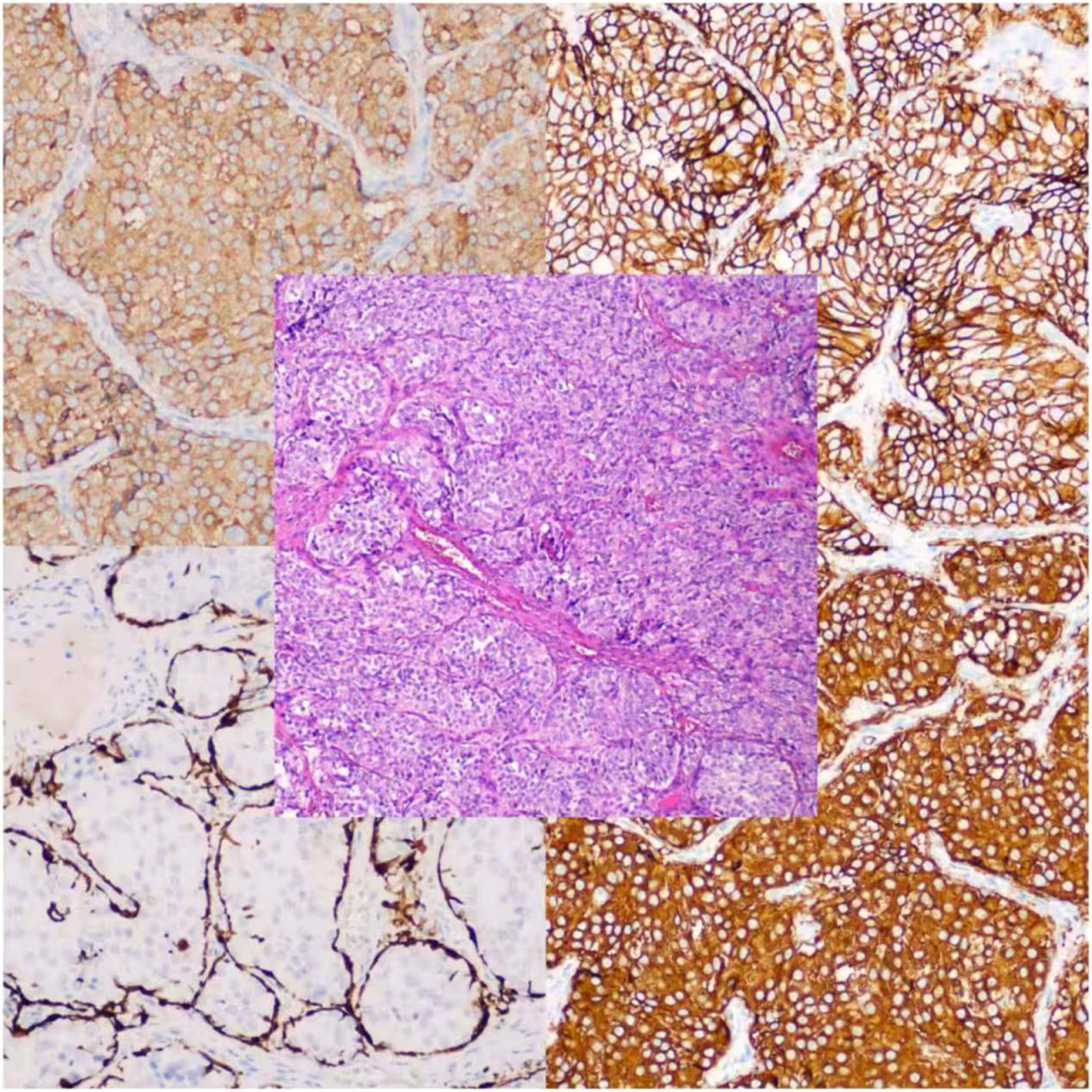
Histopathological examination with immunohistochemical staining confirmed the diagnosis of paraganglioma.

A 60-year-old woman was referred to our institution with a one-year history of progressive exertional chest tightness and dyspnea, which had significantly worsened during the preceding month. The patient had a history of poorly controlled hypertension. Physical examination revealed no remarkable cardiovascular or respiratory abnormalities. She was a teetotaler, nonsmoker and never abused any addictive drugs. Laboratory investigations demonstrated elevated urinary catecholamine levels: norepinephrine 148.4 μg/24h (reference range: 0-90 μg/24h), epinephrine 56 μg/24h (reference range: 0-20 μg/24h), and dopamine 854.2 μg/24h (reference range: 0-600 μg/24h). Chest computed tomography (CT) revealed a soft tissue tumor of approximately 50×40×30 mm in the pericardial region superior to the left ventricle (**Figure 1A**). To further characterize the lesion, cardiac magnetic resonance imaging (MRI) was performed, which confirmed a pericardial tumor in the basal left ventricular region (**Figure 1A**). The imaging characteristics were suggestive of a neurogenic tumor and were consistent with the CT findings. Coronary computed tomography angiography (CTA) showed no significant abnormalities in the origin or course of the left or right coronary arteries and the pericardial tumor was considered a neoplastic lesion and was suspected to receive blood supply from the coronary circulation (**Figure 2**). Fluorodeoxyglucose positron emission tomography (FDG-PET) demonstrated heterogeneous fluorodeoxyglucose uptake within the tumor, suggestive of a malignant neoplasm (**Figure 3**). No evidence of distant metastatic disease was identified. Given the suspicion for a malignant pericardial tumor, an ultrasound-guided percutaneous pericardial biopsy was performed. Histopathological examination revealed the classic “Zellballen” pattern—nested clusters of chief cells surrounded by a delicate fibrovascular stroma. Immunohistochemical staining was positive for chromogranin and synaptophysin in the chief cells, and showed sustentacular cells positive for S-100 protein at the periphery of the cell nests, confirming the diagnosis of paraganglioma (**Figure 4**). Surgical resection was recommended as the primary treatment. The patient declined surgical intervention due to concerns over the associated risks. Selective coronary angiography confirmed the absence of abnormalities in the origin and course of the coronary arteries and revealed no significant stenosis. Angiography revealed that a proximal branch of the circumflex artery provided blood supply to the cardiac tumor. The ostium of the left coronary artery was dilated, and a tortuous fistula, approximately 2 mm in diameter, was observed arising from the circumflex artery and communicating with the pericardial tumor (**Figure 1A**). A multidisciplinary team assessment concluded that the pericardial tumor was dependent on the circumflex artery fistula for its blood supply. Embolization of the fistula was proposed to devascularize the tumor and achieve therapeutic goals. Therefore, transcatheter embolization of the fistula was performed. The fistula was selectively catheterized under fluoroscopic guidance. A microcatheter was advanced to its distal segment. Three detachable coils were deployed through the microcatheter to occlude the fistula. Post-embolization angiography confirmed complete occlusion of the fistula with no residual flow. Post-procedural 24-hour urinary catecholamine levels normalized (norepinephrine, 42.6 μg/24h; epinephrine, 3.7 μg/24h; and dopamine, 402.7 μg/24h). At the one-year follow-up, surveillance chest CT (**Figure 1B**) and cardiac MRI (**Figure 1B**) demonstrated a significant reduction in tumor size. Follow-up coronary angiography also revealed a marked reduction in tumor size (**Figure 1B**). During follow-up, the patient reported significant resolution of her chest tightness and dyspnea, and her blood pressure was well-controlled.

## Discussion

Pericardial tumors are extremely rare, with an estimated incidence of 0.001% to 0.03% (3), primary tumors of the pericardium are rarer still. The clinical manifestations of patients are diverse, usually asymptomatic, and when symptoms appear, they may also be non-specific, including chest pain, difficulty breathing, palpitations, fainting, or systemic symptoms (4). Our patient initially presented primarily with chest tightness and shortness of breath. Pericardial paraganglioma is a slow-growing tumor derived from neural crest cells that is usually non-functional (5). Their functional counterparts are extremely rare. Although most paragangliomas are benign, some may demonstrate local invasiveness or distant metastasis. Similar to paragangliomas at other anatomical sites, functional pericardial paragangliomas may cause symptoms related to catecholamine excess, including tremor, diaphoresis, palpitations, headache, and hypertension (6). Non-functional pericardial paragangliomas may be discovered incidentally during imaging studies or may be identified when patients experience symptoms due to mediastinal structural compression. Pericardial paragangliomas most commonly occur in the left atrium or anterior to the aortic root, near the coronary artery origins. Paragangliomas are uncommonly found in the pericardium, with fewer than 200 cases reported in the literature (7). Their histological features are similar to those of extrapericardial paragangliomas. They originate from autonomic ganglia associated with the aorta, pulmonary artery, or coronary arteries. This case is clinically significant for several reasons. First, it demonstrates the critical role of comprehensive imaging—from initial CT/MRI to definitive angiography—in characterizing a pericardial mass and its vascular supply. Second, it confirms the diagnostic power of biopsy with immunohistochemistry, where the classic Zellballen histology and marker profile (chromogranin+, synaptophysin+, S100+ sustentacular cells) are pathognomonic. Third, and most importantly, it presents a successful alternative to surgery in a patient with a functional tumor fed by a coronary collateral.

Imaging plays a crucial role in the diagnosis of primary pericardial tumors. Conventional diagnostic modalities include chest radiography and transthoracic echocardiography. Characteristic imaging findings include an enlarged cardiac silhouette, abnormal mediastinal contours, or a mediastinal tumor. Although echocardiography is widely employed, its diagnostic utility for primary pericardial tumors is limited. CT optimally delineates tumor location, relationship to adjacent structures, and invasion of critical anatomical areas (8). CT also helps to narrow down the scope of differential diagnosis (8). In addition, CT can confirm the presence of local or distant metastatic diseases (9). Magnetic resonance imaging (MRI) provides superior soft-tissue contrast resolution compared to CT, making it more powerful for lesion characterization (9). Another advantage of magnetic resonance imaging is its ability to demonstrate myocardial invasion and gain a deeper understanding of the functional impact of new plasma (9). FDG-PET/CT serves as a valuable staging modality by detecting both localized and distant disease spread (10). Histopathological examination remains the gold standard for diagnosis and is crucial for determining tumor type and malignant potential. It provides essential guidance for treatment planning. In our case, multimodal imaging and histopathological examination confirmed a pericardial paraganglioma supplied by coronary collateral vessels. Since the patient declined surgical intervention, we performed transcatheter embolization of the feeding vessels. At one-year follow-up, the tumor had significantly regressed and hormone levels had normalized.

The clinical significance of embolizing a coronary collateral vessel lies in its potential to achieve a “medical devascularization.” By precisely occluding the feeding artery, the tumor is deprived of its blood supply, leading to ischemic necrosis and regression, as evidenced by the radiographic and biochemical normalization in our patient.

This report has inherent limitations. As a single-case study, its findings are not generalizable. The long-term durability of the embolization beyond one year is unknown, and the patient requires lifelong surveillance for potential tumor recurrence or revascularization via new collaterals. The procedure’s success is highly dependent on favorable anatomy, and its application in other patients would require equally careful pre-procedural planning. Future studies and case registries are needed to better define the selection criteria for embolization versus surgery and to assess long-term outcomes.

## Conclusion

The differential diagnosis of pericardial tumors is complex, encompassing non-neoplastic, benign, and malignant etiologies. The frequent absence of specific clinical features complicates early detection. A comprehensive evaluation incorporating clinical presentation, multimodality imaging, and histopathological assessment is essential for establishing accurate diagnoses and formulating appropriate management strategies. This case demonstrates the therapeutic potential of transcatheter embolization for selected patients with pericardial paragangliomas who are not surgical candidates. Finally, we summarized the characteristics of embolization and standard surgical therapy (**Table 1**).

**Table 1:** Embolization vs. surgery: a comprehensive comparison of key features.

Feature/aspect	Preoperative embolization	Primary surgical resection
Primary Surgical Resection	Reduce intraoperative blood loss and facilitate surgical dissection by inducing tumor devascularization and necrosis.	Achieve complete tumor removal for curative intent.
Primary Surgical Resection	Large or hypervascular tumors where bleeding risk is high. Tumors with complex vascular supply that can be safely accessed.	Gold standard for all resectable mediastinal paragangliomas. Necessary when tumor invades vital structures like great vessels, atria, or coronary arteries.
Key Advantages	Reduces estimated blood loss during surgery. Can decrease operative time. May facilitate complete resection by clarifying surgical planes.	Definitive, curative treatment. Allows for en-bloc resection of involved structures. Provides tissue for histological grading and genetic testing.
Key Advantages	Strictly contraindicated if tumor feeders arise from coronary arteries (high risk of MI/infarction). Risk of non-target embolization. Does not eliminate need for surgery; adds an extra procedure and cost.	High morbidity surgery, often requiring sternotomy and possible cardiopulmonary bypass Significant risk of bleeding. Nerve injury risk.
Impact on Surgery	Ideally performed 1–7 days before surgery. Tumor becomes firmer and less vascular due to ischemia and fibrosis. Improves rate of complete resection.	Requires meticulous planning with multidisciplinary team (cardiac/thoracic surgery, anesthesia). Intraoperative hemodynamic swings are common and must be managed aggressively.
Specific Complications	Acute renal failure and pneumonia associated with embolization alone. Pleuritic pain (common in first 24h). Higher complication rate if done with surgery in same admission.	Postoperative complications in ~28% of patients. Cranial nerve deficits. Atrial fibrillation. Potential for catecholamine withdrawal symptoms post-resection.
Cost & Hospital Stay	Embolization (with or without surgery) associated with significantly increased hospital-related costs.	Hospital stay median of 5–9 days. ICU stay median of 2.5 days.

## Data Availability

The original contributions presented in the study are included in the article/supplementary material. Further inquiries can be directed to the corresponding author.
